# Fabrication of Dense Non-Circular Nanomagnetic Device Arrays Using Self-Limiting Low-Energy Glow-Discharge Processing

**DOI:** 10.1371/journal.pone.0073083

**Published:** 2013-08-14

**Authors:** Zhen Zheng, Long Chang, Ivan Nekrashevich, Paul Ruchhoeft, Sakhrat Khizroev, Dmitri Litvinov

**Affiliations:** 1 Electrical and Computer Engineering, University of Houston, Houston, Texas, United States of America; 2 Chemical and Biomolecular Engineering, University of Houston, Houston, Texas, United States of America; 3 Chemistry, University of Houston, Houston, Texas, United States of America; 4 Center for Integrated Bio and Nano Systems University of Houston, Houston, Texas, United States of America; 5 Electrical & Computer Engineering, Florida International University, Miami, Florida, United States of America; Texas A&M University, United States of America

## Abstract

We describe a low-energy glow-discharge process using reactive ion etching system that enables non-circular device patterns, such as squares or hexagons, to be formed from a precursor array of uniform circular openings in polymethyl methacrylate, PMMA, defined by electron beam lithography. This technique is of a particular interest for bit-patterned magnetic recording medium fabrication, where close packed square magnetic bits may improve its recording performance. The process and results of generating close packed square patterns by self-limiting low-energy glow-discharge are investigated. Dense magnetic arrays formed by electrochemical deposition of nickel over self-limiting formed molds are demonstrated.

## Introduction

Each progressing year, the magnetic recording industry introduces new hard disk drives with ever increasing areal bit density. To sustain a healthy areal bit density growth rate, engineers and scientists around the globe are developing new magnetic recording technologies that can succeed perpendicular recording, which may reach its technological limit in as little as a few years. One contending approach is bit-patterned media, where each bit is a single magnetic grain that is defined using lithographic processes [1,2]. While optimizing the lithography process to produce bit-patterned media, we’ve developed a new process called self-limited ion milling (SLIM) that may have some exploitable advantages for bit-patterned media fabrication.

SLIM was reported by our group in 2007 as a process to produce non-circular patterns such as squares or hexagons from circular patterns in a precursor [3]. The precursor is polymethyl methacrylate (PMMA) that contains an array of circular patterns generated by electron beam lithography. When the precursor is processed in an argon ion mill, the circular patterns gradually transform into squares or hexagons depending on the array lattice. This process is potentially transformative because sharp corners are inherently difficult to produce at sub-100 nm dimensions.

Traditionally, high-resolution patterns are produced by electron beam lithography where a Gaussian-shaped beam is vector scanned to expose patterns in resist. The electron beam has a finite size, so sharp corners are inherently impossible to achieve. In addition, the proximity effect [4–8] and resist development processes further limits the practical corner radius to approximately 10 to 20 nm. Furthermore, electron beam lithography is a serial patterning technique that is not economically feasible or practical in a manufacturing environment – it is estimated that 1 month is required to pattern a single bit-patterned media disk with an areal density of 1 Tb/in^2^, which is less than twice the density of perpendicular recording in 2010. A more likely approach for bit-patterned media fabrication will involve self-assembled block copolymer systems where circular patterns are thermodynamically favorable [9,10].

In this work, we investigate the SLIM process as an inexpensive approach to fabricate non-circular device patterns. SLIM will be performed using a reactive ion etcher (RIE), which is a common, inexpensive and high throughput processing tool used in the microelectronics industry to selectively etch materials. It should be noted that the process explored here is not “reactive” ion etching but rather a version of ion milling/sputtering with low ion energy utilizing an RIE system. Due to the availability of RIE systems and the simplicity of the process, it should be relatively easy to adapt this process for manufacturing. Square patterns, in particular, have approximately 27% more surface area than a circle pattern with the same critical dimension. The extra area can benefit bit-patterned media by increasing the thermal stability [11] and signal to noise ratio of every bit. In addition, the SLIM process can be extended to magnetic random access memory (MRAM) [12] fabrication providing similar improvements as bit-patterned media.

## Experiment and Results

### 1. SLIM Process

In the SLIM process, circular openings in a PMMA resist (precursor) are transformed to non-circular patterns by low-energy argon ion bombardment. The shape of the resulting non-circular pattern depends on the array lattice. A square lattice will produce squares; a honeycomb lattice will produce hexagons. When argon ions bombard PMMA, sputtering occurs at the surface while oxygen and hydrogen are debonded in the shallow region below the surface [13,14]. This causes the composition of PMMA to change to a more etch resistant graphite. The interplay between sputtering, PMMA decomposition to graphite and the array lattice has enabled the transformation of circle patterns to square patterns.

**Figure 1:**
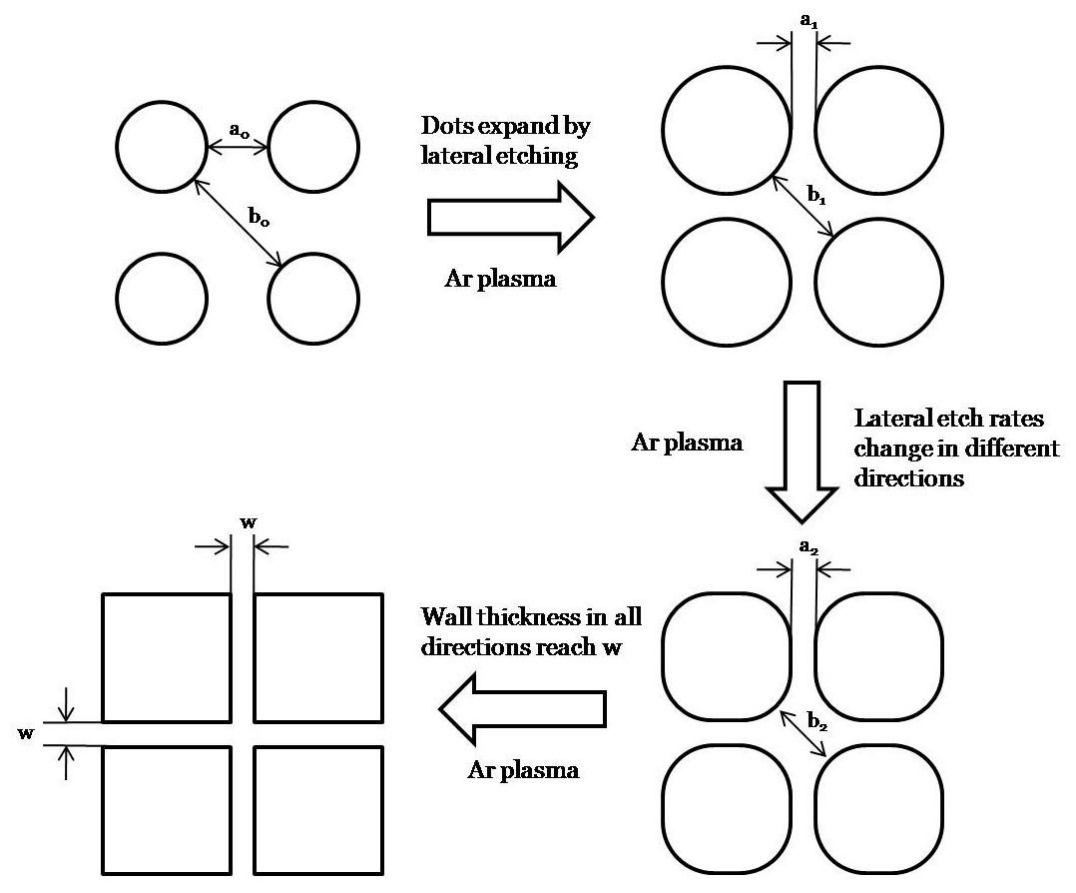
Diagram describing the SLIM process. The precursor is dot patterns separated by a wall of thickness a_0_ and b_0_. SLIM processing narrows both walls at the same rate to a thickness of a_1_ and b_1_. At a critical thickness the etch rate reduces significantly resulting in little change towards a_2_, but b_2_ continues to narrow. As b_2_ approaches the critical thickness, the pattern converges to a square.

The SLIM process is illustrated in Figure 1. The precursor is an array of circular openings formed in PMMA using electron beam lithography arranged in a square lattice. The wall thickness along the xy axis, a_0_, is thinner than the wall thickness along the diagonal, b_0_. Initially, when the pattern is etched in an argon plasma, the lateral etch rate is isotropic. When the wall thickness approaches the critical value of approximately 20 nm, the lateral etching rate drops significantly. In this regime, a_0_ approaches a_1_ and stops etching while b_0_ approaches b_1_ and continues to etch at the same rate. As the etching progresses, the initial circular pattern begins to transform into square patterns as the walls stop etching at the critical thickness. Lateral etching of the pattern stops when all the walls reach the critical thickness where the pattern is a complete square. Further etching seems to erode the pattern resulting in rounder corners.

The PMMA precursor patterns were written using an FEI XL-30 FEG Scanning Electron Microscope (SEM) equipped with NanoPattern Generation System (NPGS). The thicknesses of the PMMA were measured using a Veeco Dimensions 3000 Atomic Force Microscope. The SLIM process is performed using a commercial reactive-ion etcher (RIE), Oxford 100 Plus. The RIE is pumped down to a base pressure of 3.75 × 10^-5^ Torr. Argon is injected into the chamber at a rate of 15 sccm and a process pressure of 10 mTorr is maintained throughout the process. The RF power was set to 70 W resulting in DC bias of 250 V. The low energy of ions is desirable to improve the selectivity between PMMA and graphite and avoid etching of substrate, which can lead to increase of redeposition rate.

**Figure 2:**
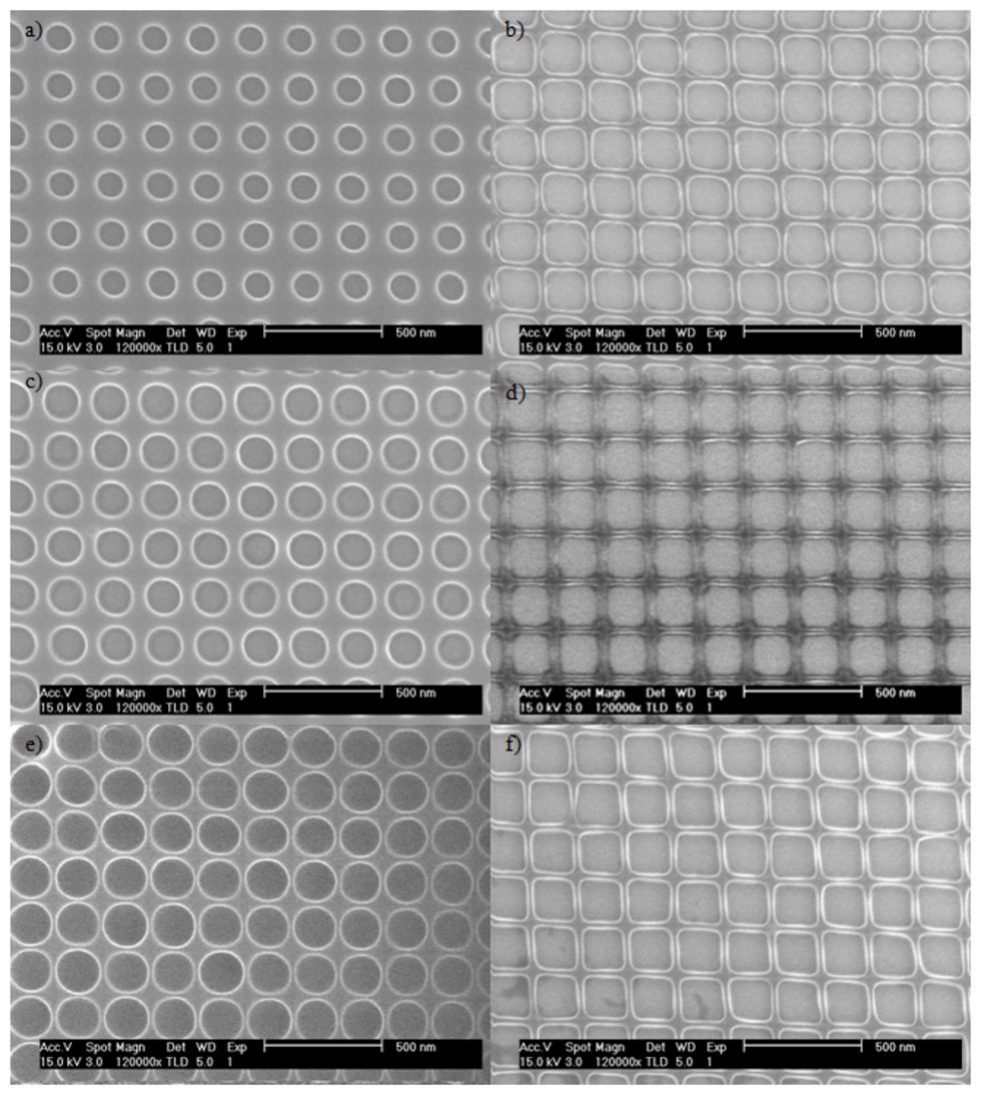
SEM images of the original precursors (a,c,e) and after SLIM processing (b,d,f). The diameters of the circles in the precursor are: a) 100nm, c) 137nm and e) 175nm.

**Figure 3:**
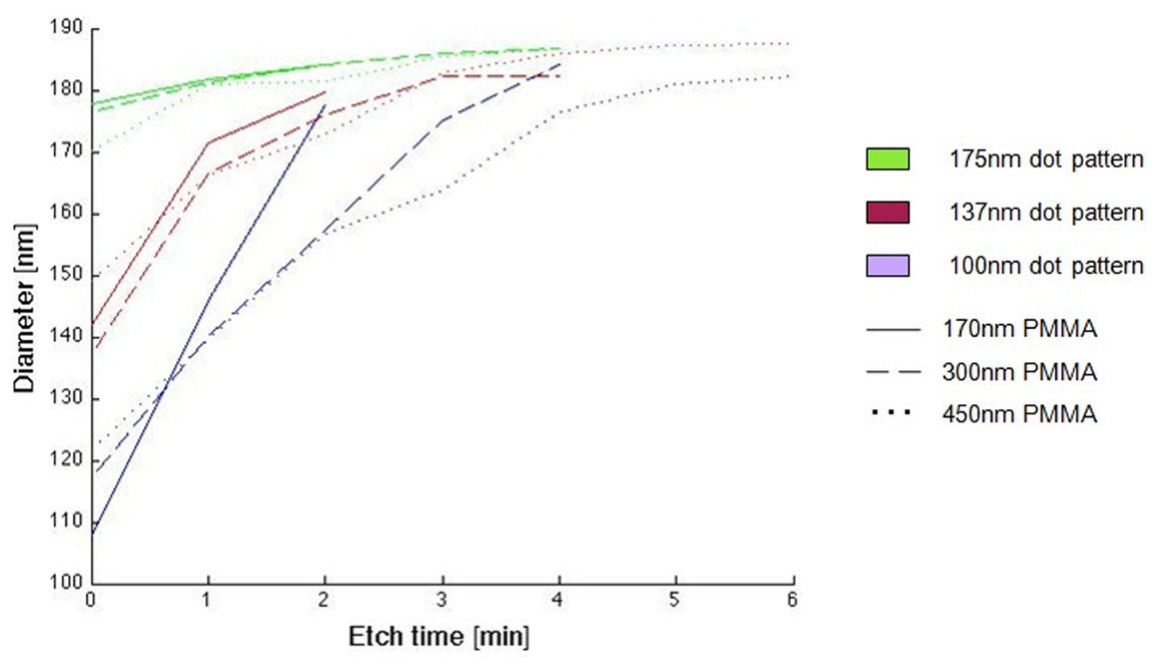
A plot showing the influence of SLIM process time and precursor thickness on the pattern width. Each curve approaches an asymptote at approximately 183nm revealing the self-limiting etch process.

To investigate the process characteristics, the SLIM process was applied to precursor patterns of circular openings of 100 nm, 137 nm, and 175 nm in diameter arranged on a 200 nm pitch square grid. PMMA thickness of 170 nm, 300 nm and 450 nm was used. The pattern morphologies before and after SLIM processing are compared in Figure 2. Figures 2a, 2c, and 2e are the precursor dot patterns in 300 nm thick PMMA with diameters of 100 nm, 137 nm and 175 nm respectively. Figure 2b, 2d, and 2f are their corresponding square patterns after 3 minutes of SLIM processing. Although the original dot diameters are different among the three precursors, the resulting squares are approximately the same size. The final wall thickness of nearby square pattern is approximately 20 nm, and it does not depend on the initial size of the precursor pattern. The progression of the pattern widths as a function of etching time is shown in Figure 3. It is evident from Figure 3 that the precursor dot diameter and the initial PMMA thickness influence the success of the process, but it does not dictate the shape of the resulting pattern. During SLIM, the resist is etched both laterally and vertically. When the resist is too thin and the dot size is small, the resist gets completely etched before square patterns can form. On the other extreme, using thick resist can impede the SLIM process when the initial dot diameter is too small. Between these two extremes, there is a significant processing window where square patterns can be readily achieved as evidenced by the many curves in Figure 3 that approached the asymptote at approximately 182 nm.

### 2. Data analysis

**Figure 4:**
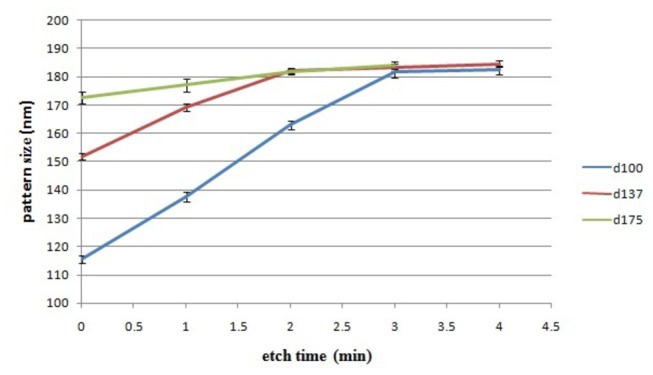
A plot of pattern size vs etch time for different precursor openings showing little change in the pattern size distribution.

Statistical analysis was performed to monitor the size and shape distribution of the SLIM patterns at fixed time intervals. The experiment was carried out on precursors of 300 nm thick PMMA with dot sizes of 100/137/175 nm on a 200 nm square grid. The time dependence of the pattern size and size distribution is shown in Figure 4. The trend in Figure 4 is the same trend as Figure 3, confirming the reproducibility of our experiments. The pattern size distribution ranges from approximately 1.5% to 2.5% as portrayed by the error bars in Figure 3. It is noticed that SLIM process has limited influence on improvement of pattern size distribution for the tested precursor conditions. The SLIM process etches laterally until a critical wall thickness where the etch rate slows down significantly. Therefore, the position of the wall should depend greatly on the size and position of the neighboring patterns.

**Figure 5:**
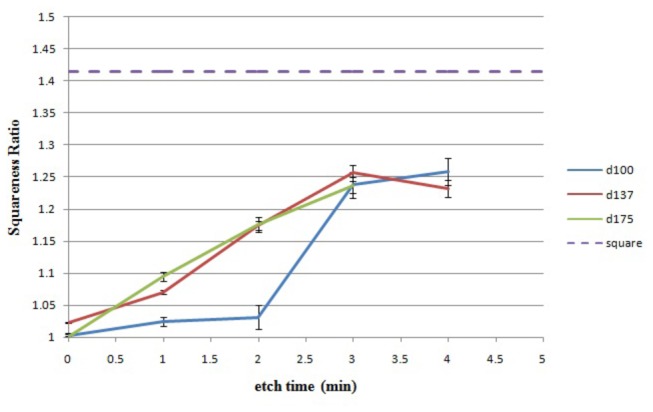
A plot of the measured squareness ratio vs etch time for each precursor.

**Figure 6:**
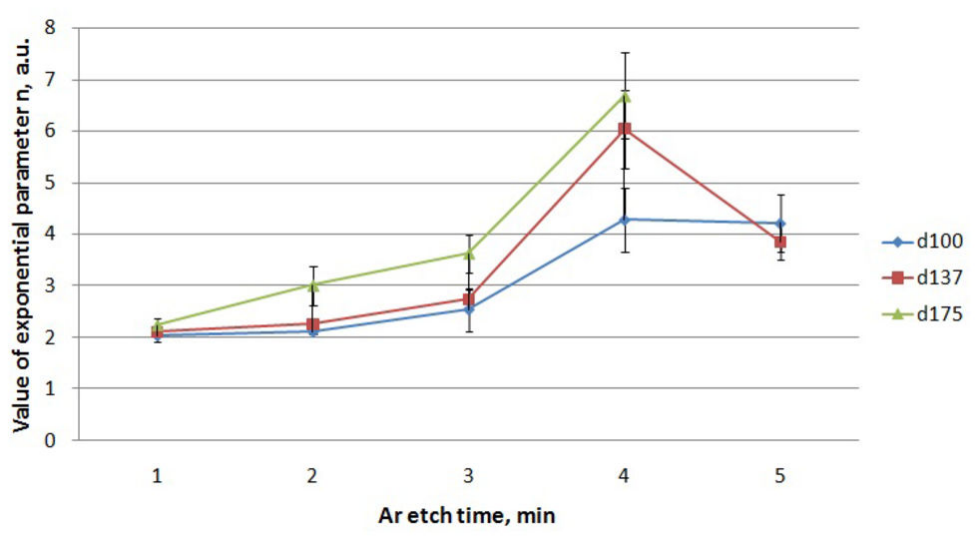
A plot of the optimized squareness value, *n*, vs etch time.

The shape of the pattern is characterized by a squareness ratio that is calculated by dividing the length of the diagonal by the width of the pattern. The dependence of the squareness on the etching time for the set of precursors is shown in Figure 5. As a reference, the dashed line represents the squareness ratio of a perfect square and the squareness ratio of a circle is 1. For the precursor with 100 nm dots, the transformation from a circle to a square is relatively slow for the first 2 minutes. The slow transformation is because it takes longer time for smaller circles to grow to the point where shape expanding is influenced by nearby patterns. After 2 minutes, the transformation proceeds rapidly and then stops at a squareness ratio of approximately 1.25. The precursor with the 137 nm and 175 nm dots have similar curves showing a constant rate of change until it stabilizes at a maximum squareness ratio of 1.25 as well. For all precursors, additional processing after the transformation will simply sputter the resist with negligible changes to the pattern shape until the resist is gone. To quantify evolution of the pattern shape during argon milling we used in-house developed Python code to fit the contours of the SEM image of the pattern at different milling times with a curve of the following function:

$$k{\left|x-x_{c}\right|}^{n}+{\left|y-y_{c}\right|}^{n}=r^{n},$$

where *k* represents stretching or contraction along *x*-axis, *r* is a half length of a contour’s side, and *x*_*c*_ and *y*_*c*_ are the coordinates of the center of the contour on the image. Larger values of the parameter *n* corresponds to a shape approaching a rectangle; at *k* = 1 and *n* = 2, the shape is a circle. These five parameters are optimized simultaneously to the contours of the SEM image. Before argon milling, the shape of the printed precursors is close to a perfect circle with a *n* approximately 2. As the argon milling time increases, the circular precursor patterns gradually transforms into squares with rounded corners resulting in higher values of *n*, as shown in Figure 6. Precursors with an initial diameter of 100nm and 137nm demonstrate a decrease of growth rate of *n* or even a decrease of *n* after 3 minutes of milling. The later phenomena can be explained by over-etching of the resist resulting in an overall pattern deterioration. The maximum value of *n* is approximately 6.7 for the 175 nm dot pattern etched for 3 minutes. The 137 nm dot pattern reached an *n* value of 6, while 100 nm dot pattern reached an *n* value of 4.2. According to our calculations, a squareness ratio of 1.25 is equivalent to an *n* value of 6. This confirms the validity of the extracted *n* values and supports that either method is a reasonable way to characterize squareness. Since there is more contrast between the extracted *n* values, Figure 6 reveals that a precursor with dot sizes of 137 nm and 175nm yields squares with sharper corners than the precursor with the 100nm dots. There is little difference between the results of the 137 nm and 175 nm precursor, but the 137 nm precursor can be produced approximately 39% faster than the 175 nm precursor via electron beam lithography. In other words, a precursor with a diameter to pitch ratio of approximately 2/3 is optimal for the SLIM process.

**Figure 7:**
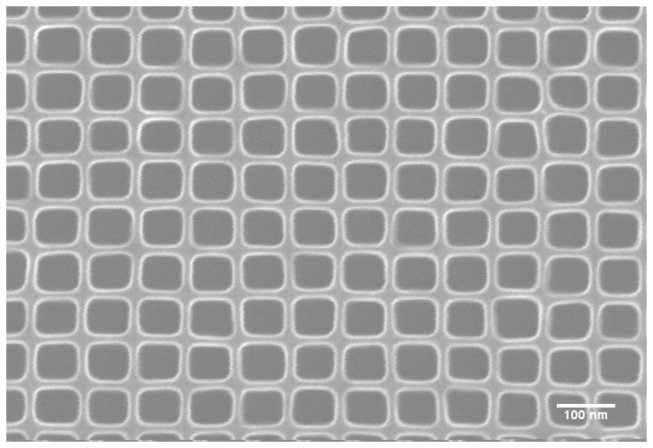
SEM image of a 75nm wide square pattern fabricated by SLIM processing of a dot array with 100nm pitch.

According to this SLIM effect, non-circular patterns of different shapes and sizes can be produced based on the precursor design. Well packed 75 nm square pattern formed by argon etching on a PMMA layer from a precursor of dot array with 100 nm pitch is shown in Figure 7. This square pattern has a size distribution less than 1% and a squareness ratio of 1.22.

### 3. Thermal stability of SLIM patterns

**Figure 8:**
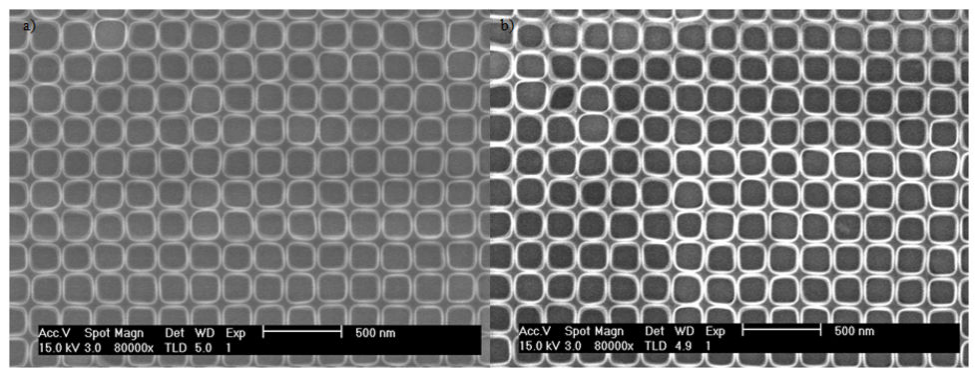
The SEM images compares a) a SLIM processed pattern and b) the same sample after heat treatment for one hour at 400 °C.

Untreated PMMA has a glass-transition temperature of 109° C. When patterned PMMA is heated beyond its glass-transition temperature the resist reflows resulting in significant degradation of the patterns. Since SLIM processing is expected to change the composition of the resist from PMMA to a more graphite-like material, the heat resistance of the processed resist is expected to improve. The effects of heat treatment on patterned PMMA and SLIM processed PMMA was investigated by heating the samples in an Accu Thermo AW410 Rapid Temperature Processing (RTP) at temperatures ranging from 100 to 400 °C. The effects of a 1 hour heat treatment at 400 °C on SLIM processed PMMA patterns are shown in Figure 8. After the heat treatment, the patterned PMMA patterns show significant degradation while the SLIM processed PMMA pattern looks unchanged. Additionally, the squareness ratio before and after the heat treatment of the SLIM processed PMMA pattern was 1.214 and 1.215 respectively, confirming negligible change in the pattern shape. The significant enhancement of the heat resistance of the SLIM processed PMMA patterns supports the theory that low energy ion bombardment modifies the chemical composition of PMMA.

### 4. Pattern Electrochemical Deposition

Electrochemical deposition was used to deposit nickel magnetic nanostructures into the SLIM processed patterns. This experiment enables a profile view of the morphology of SLIM templates [15]. The substrate used for this experiment is an N^+^-doped Si (100) wafer coated with a Ta(5nm)/Pd(10nm) seed layer was used as the substrate. It is important to strip the insulating native oxide by immersion in a 10% buffered hydrofluoric acid (HF) followed by rinsing in de-ionized water prior to seed layer deposition. The electrolyte is a solution of 0.75M NiCl_2_. Electrochemical deposition is carried out in a Gamry PC4/750 potentiostat configured to generate a 10 Hz square wave voltage pulse from -675 mV to 325 mV. The delay between deposition pulses allows the locally depleted Ni ions to replenish enabling a higher quality deposition. Following the deposition of nickel into the openings in the SLIM processed patterns, the resist is stripped by reactive ion etching with an oxygen plasma.

**Figure 9:**
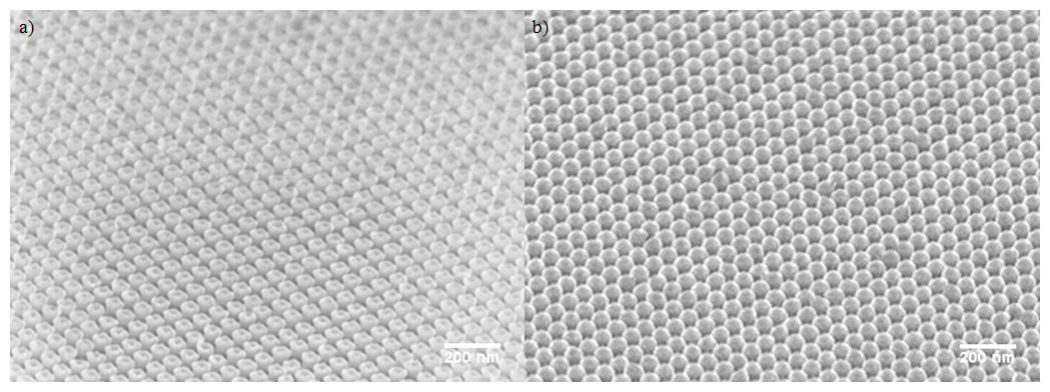
Nickel was electrodeposited into a SLIM pattern with 80nm square opening. The SEM images show a) a 52 degree projection of the nickel pillars after removing the resist and (b) a 52 degree projection of the same sample after argon plasma treatment.

Projected SEM images, 52 degrees tilt, of the electrodeposited nickel pillars after stripping the resist are shown in Figure 9. 80 nm square Ni pillars formed by stopping the electro-deposition before overgrowth occurs are shown in Figure 9a. The indent in the center of each Ni pillar resembles the filling issue that occurs during conformal deposition inside the trench – growth along the sidewalls eventually merges resulting in a dimple at the center of the pattern. This observation suggests that the SLIM processed resist is conductive [16]. Attempts to etch the pattern in an argon plasma to remove the overgrowth or flatten the dimple have always resulted in the degradation of the square shape of the Ni pillars, as shown in Figure 9b.

## Conclusions and Future Work

A new way of fabricating non-circular nanostructure is demonstrated. This SLIM process transforms circular patterns that are pre-defined by electron beam lithography in PMMA resist into non-circular patterns such as squares simply by dry etching with an argon plasma. This process was discovered in a custom-built argon mill, but it has been successfully adapted to a commercial reactive ion etcher. While the process is not completely understood, we have experimentally determined some conditions that are required for optimal squareness. This process may enable the production of sub-100 nm non-circular patterns that is inherently difficult with electron beam lithography due to the proximity effect.

Our experiments show that the initial diameter of the precursor should be approximately 2/3 of the pitch. The resist thickness for the precursor should be thicker than the pitch, but this should be studied more deliberately in the future. Two methods were devised to characterize the squareness and they both agree that the maximum squareness has corners with a radius of curvature of approximately 20 nm. The resulting SLIM patterns have straight sidewalls and minor faceting, even though the pattern is processed in a pure argon plasma. We’ve also observed from thermal treatment and electro-deposition that the SLIM process chemically changes PMMA to a material that is conducting and thermally stable at 400 °C.

Future research will be focused on further optimizing the fabrication process towards higher density patterns and investigating its application in bit patterned media. Specifically, it will be interesting to explore the possible advantages of a square bit versus a circular bit such as higher thermal stability and higher moment per bit.
